# Seasonal influenza vaccination coverage and its determinants among nursing homes personnel in western France

**DOI:** 10.1186/s12889-017-4556-5

**Published:** 2017-07-07

**Authors:** Christelle Elias, Anna Fournier, Anca Vasiliu, Nicolas Beix, Rémi Demillac, Hélène Tillaut, Yvonnick Guillois, Serge Eyebe, Bastien Mollo, Pascal Crépey

**Affiliations:** 1 Ecole des Hautes Etudes en Santé Publique, Université Sorbonne Paris Cité, Rennes, France; 2 Ecole Pasteur-CNAM de Santé Publique, Paris, France; 3 Epiter, F-94415 Saint-Maurice, France; 4 Santé publique France, Cellule d’intervention en région Bretagne, F-94415 Saint-Maurice, France; 5 UMR “Emergence des Pathologies Virales”, Aix-Marseille University - IRD 190 - Inserm 1207 - EHESP, Marseille, France; 6 EA 7449 Reperes, EHESP - Université de Rennes 1, Rennes, France

**Keywords:** Influenza vaccination, Nursing homes, Vaccination coverage, Cross-sectional study, France

## Abstract

**Background:**

Influenza-associated deaths is an important risk for the elderly in nursing homes (NHs) worldwide. Vaccination coverage among residents is high but poorly effective due to immunosenescence. Hence, vaccination of personnel is an efficient way to protect residents. Our objective was to quantify the seasonal influenza vaccination (IV) coverage among NH for elderly workers and identify its determinants in France.

**Methods:**

We conducted a cross-sectional study in March 2016 in a randomized sample of NHs of the Ille-et-Vilaine department of Brittany, in western France. A standardized questionnaire was administered to a randomized sample of NH workers for face-to-face interviews. General data about the establishment was also collected.

**Results:**

Among the 33 NHs surveyed, IV coverage for the 2015–2016 season among permanent workers was estimated at 20% (95% Confidence Interval (CI) 15.3%–26.4%) ranging from 0% to 69% depending on the establishments surveyed. Moreover, IV was associated with having previously experienced a “severe” influenza episode in the past (Prevalence Ratio 1.48, 95% CI 1.01–2.17), and varied by professional categories (*p* < 0.004) with better coverage among administrative staff. Better knowledge about influenza prevention tools was also correlated (*p* < 0.001) with a higher IV coverage. Individual perceptions of vaccination benefits had a significant influence on the IV coverage (*p* < 0.001). Although IV coverage did not reach a high rate, our study showed that personnel considered themselves sufficiently informed about IV.

**Conclusions:**

IV coverage remains low in the NH worker population in Ille-et-Vilaine and also possibly in France. Strong variations of IV coverage among NHs suggest that management and working environment play an important role. To overcome vaccine “hesitancy”, specific communication tools may be required to be adapted to the various NH professionals to improve influenza prevention.

**Electronic supplementary material:**

The online version of this article (doi:10.1186/s12889-017-4556-5) contains supplementary material, which is available to authorized users.

## Background

Seasonal influenza epidemics are a major public health problem worldwide, increasing annually the morbidity and mortality burden in vulnerable populations, particularly children younger than 2 years old and adults aged 65 years and older. Structures that concentrate vulnerable populations such as nursing homes for the elderly (NHs) or other long-term care facilities require special public health attention [1, 2]. Despite a high coverage of influenza vaccination (IV) among NH residents, influenza remains a major cause of death in this population [3].

Elderly people are poorly protected by vaccination due to immunosenescence [4]. Hence, prevention of influenza cases relies mainly on preventing infection among their direct contacts – an indirect protection strategy. In NHs, preventing influenza cases and deaths depends on preventing the virus from entering the community via the vaccination of healthcare workers (HCW) and other NH workers [5, 6]. Consequently, improving NH workers’ IV rate is a major public health target to alleviate the morbidity and mortality burden of influenza in the elderly population. In fact, 10% to 30% of HCW are infected with influenza each winter [7, 8], and most continue to work [9] which may lead to transmission of the virus to patients. Moreover, immunizing NH workers has been shown to decrease influenza transmission which increases the benefits of vaccination [5, 6].

IV of at least 75% of personnel is recommended by World Health Organization (WHO), CDC and the French Ministry of Health [10, 11]. In the US, various interventions performed in long-term facilities have contributed to an increase in HCW IV coverage from 36% in 2003 [12] to 86.4% in 2015 [13]. In France, HCW IV coverage, far from the WHO target, was 33.6% in 2008 and 25.6% in 2009 [3, 7, 8, 14–18].

In order to globally improve NH workers’ IV coverage, it is important to understand the determinants driving the vaccination status, particularly in settings with low coverage. The primary objective of the study is to estimate the IV coverage for the 2015–2016 winter season in NH workers in Ille-et-Vilaine, a department of the Brittany region*,* located in western France. Secondary objectives aim to assess the factors related to IV among NH workers.

## Methods

### Study design and population

**Fig. 1:**
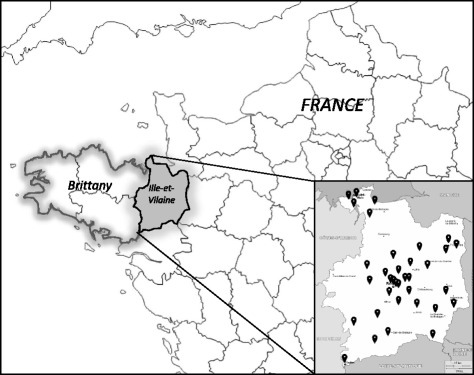
Map of the 33 nursing homes surveyed in the Ille-et-Vilaine department

A cross-sectional study was conducted among NH workers in French NHs in Ille-et-Vilaine (Fig. 1). The study took place on March 22nd 2016 and was performed as a collaboration between the French School of Public Health and Santé Publique France, in association with the regional health authority of Brittany. We performed a two-step randomized sampling, by randomly selecting NHs, then participants among the NHs. A sample size of 640 participants was computed based on an IV coverage estimation of 35%, an acceptance rate of 80%, an alpha risk of 5%, a study power of 80%, and a clustering effect. We randomly selected 40 NHs among the 137 NHs located in Ille-et-Vilaine and secondly, 16 NH workers were randomly chosen from each nursing home. Only permanent staff present at the time of the survey between 9 am and 7 pm were included, whereas temporary staff, interns, and liberal practitioners were excluded. No distinction between full time or part time workers was undertaken. In our study, NH workers included HCW such as physicians, nurses or pharmacists, administrative staff as well as facility and logistic staff. In order to reduce refusals, a letter introducing the survey was sent two weeks before the survey to the selected NHs.

### Data collection

Data was collected through two standardized and pre-tested questionnaires given as Additional files: 1, 2, 3 and 4. Questionnaire were prepared in workgroups over a two-day period where outcomes, analysis plan and variables were defined. Questions to NH managers and NH workers were then drafted and both questionnaires were tested on a sample of 1 NH manager and 5 NH workers (3 physicians and 2 nurses). The first questionnaire was addressed to NH managers to obtain information about their establishment. We gathered NH status, size and information about vaccination (information or campaign) of the NH workers. The second questionnaire, targeting NH workers, was divided in three main parts: the first focused on socio-demographic items such as gender, age, occupation and experience in a NH; the second questioned NH workers on influenza risk factors and IV history; and finally, the third part gathered information concerning knowledge about vaccination as well as the perceived benefits and barriers of influenza vaccination. The total duration of the survey lasted no more than five minutes. Both questionnaires were administered in their original language (French). All questions were closed but interviewees were offered the possibility to give final comments or remarks. Questionnaires were administered face-to-face to NH workers in the participating NHs. All interviews were realized by 31 trained investigators as part of the IDEA international field epidemiology training course.

### Data analysis

Vaccine coverage was estimated from data obtained from the interviews with the personnel. IV coverage was defined as the proportion of vaccinated NH workers for the 2015–2016 winter season. We first conducted a univariate analysis using Chi2 and Fisher exact tests to determine which determinants were significantly associated with vaccine coverage. All factors with *p* values lower than 0.2 were integrated in a multivariate Poisson model with and without random effects. Prevalence ratios (PR) and their 95% confidence intervals (CI) were used as measures of association. A *p* value lower than 0.05 was considered to be statistically significant. Data were centralized with WEPI software (Epiconcept) and analyses were performed using Stata 13® (StataCorp, Texas, USA).

## Results

### Participation

**Fig. 2:**
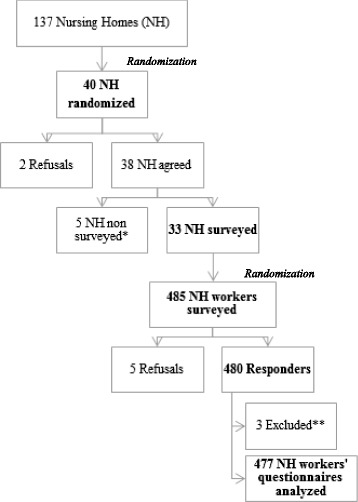
Study sample flow chart. *Late arrival or acute gastro enterities epidemic in NH. **We excluded three responders who were not matching inclusion cirteria

Of the 40 NHs randomized among the 137 in the administrative district, 33 (85%) participated in this survey. Two refused and 5 could not be visited for logistic reasons. Of the 485 personnel surveyed, 480 answered and 5 refused principally due to lack of time (response rate 99%) (Fig. 2). We excluded three responders who did not match inclusion criteria to finally include a total of 477 NH workers in our study.

### Characteristics of nursing homes and workers

Among NHs, 52.5% were public versus 47.5% with a private status, with a median number of 71 beds (min 28; max 270) (Table 1). The median number of permanent personnel was 43 (min 23; max 111) and the median ratio employee/resident was 0.73 (min 0.42; max 1.33).

**Table 1:** Population according to the nursing home characteristics

			Bivariate analysis with random effect		
Factors	Proportion of the personnel (%)	95% CI	Vaccination coverage (%)	95% CI	*p*-value
Institution type					
Status of the establishment					0.34
Public establishment	52.5	[48.1–56.9]	25.5	[14.5–32.8]	
Private establishment	47.5	[43.2–51.9]	17.5	[12.5–24.1]	
Vaccination campaign					0.93
Working in an institution without campaign	16.3	[7.6–31.7]	19.4	[6.6–45.2]	
Working in an institution with campaign	83.6	[68.3–92.4]	20.3	[15.4–26.2]	

Concerning the IV campaign, 81.8% of NH managers declared they had given information and offered the possibility of vaccination to the NH workers. Of the total study sample, 87.0% of NH workers were female. The mean age was 41 years (min 20; max 66), and they worked in a NHs for an average of 11.5 years (min 0.6; max 38). Most of them were HCW (42.9%) or facilities and logistics staff (35.7%). Administrative positions were held by 11% of workers. Overall, 84.1% of NH workers declared to be in contact with residents more than once per day.

### Influenza vaccination coverage among workers

Of all NH workers, 20.0% [95% CI 15.3%–26.4%] declared having received the IV during the 2015/16 season, among which 80% have been vaccinated within the establishment. 21.3% [95% CI 16.6%–27.3%] of them reported being regularly vaccinated, as defined by at least two vaccinations during the last three years.

**Fig. 3:**
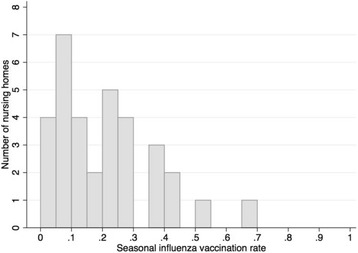
Distribution of the nursing homes according to their rate of seasonal influenza vaccination

Median IV coverage for all sampled establishments for the season 2015–2016 was estimated at 18.2% ranging from 0% to a maximum of 69.2% (Fig. 3).

### Predictors of influenza vaccine uptake

#### Socio-demographic, influenza risk factors, and vaccine history predictors

Vaccinated NH workers were older than the non-vaccinated (Table 2). Statistical analysis showed that personnel aged above 30 years were more vaccinated than personnel between 20 and 29 years (*p* < 0.01). A lower vaccine coverage was observed among the facilities and logistic staff members relative to the HCW (*p* < 0.004). Furthermore, having experienced a “severe” influenza infection (defined as “*you had to be bedridden*”) was positively associated with a higher vaccination rate (*p* < 0.045) (Table 2).

**Table 2:** Characteristics of the NH personnel and their seasonal IV coverage

			Bivariate analysis with random effect			Multivariate analysis with random effect^a^		
Characteristics	Proportion of the personnel (%)	95% CI	Vaccination coverage (%)	95% CI	*p*-value	PR	95% CI	*p*-value
Socio-demographics								
Sex					0.31			
Male	13.0	[10.0–17.0]	24.6	[15.4–36.7]				
Female	87.0	[83.2–90.0]	19.5	[14.5–25.7]				
Mean age					0.008			
Age: 20–29 yrs	17.8	[14.2–22.2]	7.0	[3.6–13.0]		ref.	–	
Age: 30–39 yrs	28.6	[24.2–33.5]	21.6	[15.0–30.1]		2.95	[1.51–5.76]	0.003
Age: 40–49 yrs	29.6	[25.9–33.6]	26.3	[18.0–36.6]		3.45	[1.86–6.41]	<0.001
Age: 50–59 yrs	20.9	[17.0–25.4]	20.3	[12.2–31.9]		2.81	[1.28–6.16]	0.012
Age: 60–69 yrs	3.1	[1.9–5.1]	26.8	[11.8–49.9]		2.91	[1.02–8.34]	0.043
Mean institution working length					0.26			
Working length: 0–5 yrs	26.2	[21.1–32.1]	15.1	[7.6–27.8]				
Working length: 5–10 yrs	31.6	[26.6–37.2]	15.8	[8.3–27.8]				
Working length: 10–15 yrs	18.6	[15.4–22.4]	27.3	[18.3–38.6]				
Working length: 15–20 yrs	14.6	[11.1–19.0]	21.8	[13.4–33.5]				
Working length: >20 yrs	8.9	[6.5–12.1]	25.1	[13.7–41.2]				
Occupation classification					0.004			
Healthcare workers	42.9	[37.7–48.2]	24.9	[18.4–32.6]		ref.	–	
Administrative	11.0	[8.5–14.1]	31.0	[18.5–47.0]		1.07	[0.74–1.55]	0.86
Facilities and logistics	35.7	[31.0–40.6]	13.1	[8.3–20.2]		0.53	[0.36–0.77]	0.003
Other	10.5	[7.8–14.1]	14.2	[6.7–27.7]		0.49	[0.25–0.98]	0.045
Contacts with residents								
Frequency of contacts with residents					0.96			
Rarely (<=1/day)	15.6	[12.2–20.6]	20.4	[11.1–34.5]				
Several times/day (>1/day)	84.1	[79.4–87.8]	20.1	[15.3–25.9]				
Influenza vaccine risk factors								
Living with children <5 yrs	23.0	[18.9–27.7]	21.7	[14.0–32.1]	0.9			
Living with elderly person >65 yrs	4.4	[2.7–7.1]	19.0	[7.6–40.2]	0.9			
Living with someone with chronic illness	8.3	[6.0–11.3]	30.6	[18.4–46.4]	0.033			
Living with someone with influenza risk factor (total)	31.6	[27.3–36.3]	25.2	[17.5–34.9]	0.09			
Having a personal medical indication to vaccination	9.5	[7.3–12.5]	26.5	[14.9–42.6]	0.33			
Influenza history								
Previous “severe” influenza infection	39.1	[35.8–42.5]	27.7	[19.8–37.3]	0.003	1.48	[1.01–2.17]	0.045

^a^Adjusted on the number of permanent worker, the number of prevention tools spontaneously mentioned and the knowledge of frequency of the vaccination

#### Knowledge about influenza

About NH workers’ knowledges of influenza and IV, 75.0% of them spontaneously mentioned hand washing as a prevention tool to avoid influenza transmission (Table 3). Wearing a mask and gloves was listed by 49.5% of NH workers, preventing contacts by 27.6% and vaccination by 24.4%. Knowledge of recommended prevention tools against transmission of influenza viruses was significantly associated with IV coverage. Spontaneous citation of one or more prevention tools was positively associated with a higher rate of vaccination (*p* < 0.001). An increasing association between the number of tools spontaneously cited and IV coverage was observed (*p* < 0.001). Moreover, 9.2% of NH workers reported homeopathy and 26.4% pointed out other tools not recommended by the national health authorities.

**Table 3:** NH personnel knowledge and perception on influenza and the seasonal IV coverage

			Bivariate analysis with random effect			Multivariate analysis with random effect^a^		
Factors	Proportion of the personnel (%)	95% CI	Vaccination coverage (%)	95% CI	*p*-value	PR	95% IC	*p*-value
Knowledge to prevent influenza transmission								
Number of prevention tools spontaneously mentioned					<0.001			
0 prevention tool	10.0	[7.7–13.0]	2.2	[0.3–14.0]		ref.	–	
1 prevention tool	26.9	[23.0–31.3]	15.1	[9.0–24.1]		6.48	[1.18–35.57]	0.034
2 prevention tools	40.7	[35.7–45.8]	21.2	[15.8–28.0]		7.75	[1.21–49.70]	0.037
3 prevention tools	21.1	[17.6–25.1]	32.3	[21.2–45.8]		9.27	[1.51–56.67]	0.022
4 prevention tools or more	1.3	[0.5–3.0]	33.3	[7.3–76.2]		11.03	[1.27–96.24]	0.039
Vaccination	24.4	[20.2–29.2]	43.5	[30.9–57.0]	<0.001			
Hands washing	75.0	[70.5–79.1]	21.5	[16.1–28.1]	0.20			
Wearing mask / gloves	49.5	[43.6–55.5]	22.2	[16.1–29.8]	0.27			
Prevent the contacts	27.6	[22.1–33.9]	20.7	[14.0–29.4]	0.86			
Other tools spontaneously mentioned to prevent influenza transmission								
Homeopathy	9.2	[6.0–13.9]	9.4	[3.4–23.5]	0.06			
Anti-viral therapy	0.2	[0.0–1.5]	0.0		0.07			
Do not know	4.4	[2.6–7.4]	19.7	[14.2–26.7]	0.06			
Knowledge of the populations at risk								
Number of population at risk spontaneously mentioned					0.15			
2 populations cited or less	69.7	[64.3–74.7]	18.2	[13.3–24.4]				
3 populations cited or more	30.3	[25.3–35.7]	24.6	[16.4–35.2]				
Elderly person >65 yrs	95.8	[93.6–97.3]	20.4	[15.3–26.6]	0.59			
Infants and young children	66.4	[61.9–70.6]	20.0	[14.0–27.6]	0.1			
Person with chronic illness	43.9	[39.4–48.5]	26.4	[20.0–34.1]	<0.001			
Pregnant women	3.4	[2.1–5.3]	6.4	[0.8–35.8]	0.16			
Overweight person	0.0	–	–					
Do not know	1.6	[0.8–3.2]	13.3	[1.8–56.2]	0.63			
Knowledge of frequency of the vaccination								
Knowledge of frequency of the vaccination					<0.001			
Other or do not known	26.1	[21.5–31.3]	4.8	[2.3–9.7]				
Every year	73.9	[68.7–78.5]	25.5	[19.1–33.2]		4.27	[1.96–9.31]	<0.001
Information channels for vaccination								
By the NHs	76.3	[69.4–82.1]	20.8	[15.5–27.4]	0.52			
By media	67.5	[62.5–72.2]	19.8	[14.0–27.3]	0.81			
By general practitioner	11.4	[8.9–14.5]	33.7	[21.1–49.2]	0.005			
By occupational practitioner	5.7	[3.6–9.1]	36.8	[22.2–54.3]	0.009			
Other	12.4	[9.3–16.3]	–					
Sensitivity to information								
Theses information’s influenced the choice	13.7	[10.3–18.0]	56.6	[41.0–71.0]	<0.001			
Information seems sufficient	78.4	[72.7–83.1]	21.1	[15.8–27.6]	0.32			
Sensitive to posters	39.7	[34.4–45.2]	–					
Sensitive to email, mail	27.9	[23.8–32.5]	–					
Sensitive to meetings and formations	52.9	[47.6–58.0]	–					
Sensitive to other information	12.5	[9.2–16.9]	–					

^a^Adjusted on the age, the number of NH personnel, the working length in an institution and the previous “severe” influenza infection

Finally, 73.9% of employees knew that IV needs to be administrated every year. And, knowledge of the frequency of the vaccination, known by a large part of the employees, was positively associated with a higher vaccination rate (*p* < 0.001). (Table 3).

#### Information

The main channels of information about seasonal IV received by the NH workers were primarily communication within the establishment and secondly communication by the media (Table 3). We also observed that 53.7% of the administrative employees were sensitive to posters as a medium. There were 51.9% of the HCW and 55.1% of the facilities and logistics staff that stated being more receptive to meetings and trainings (Table 4).

**Table 4:** NH personnel information type preference

	Sensitivity to information					
Factors	Sensitive to posters (%)	95% CI	Sensitive to email, mail (%)	95% CI	Sensitive to meetings and formations (%)	95% CI
Occupation classification						
Healthcare workers	40.7	[34.4–47.3]	29.0	[23.1–35.6]	51.9	[44.4–59.3]
Administrative	53.7	[41.0–66.0]	29.0	[17.7–43.6]	36.6	[21.9–54.3]
Facilities and logistics	36.8	[29.7–44.6]	29.5	[23.6–36.2]	55.1	[46.5–63.5]

#### Perceived benefits of influenza vaccination

Perceived benefits and barriers to seasonal IV are shown in Table 5. Among the employees, 73.7% agreed that being vaccinated protects the NH residents. This proportion is also high for the non-vaccinated NH workers (74.1%). Otherwise, 52.9% NH workers agreed with the fact that being vaccinated prevents oneself from getting influenza.

**Table 5:** NH personnel perceived benefits and barriers of seasonal influenza vaccination 2015–2016

Factors	Proportion of the personnel (%)	95% CI	Vaccination coverage (%)	95% CI
Perceived benefits of vaccination				
Getting the vaccine will prevent me from getting influenza	52.9	[47.9–57.8]	32.4	[24.8–41.1]
When you are vaccinated, you protect your entourage	68.4	[62.6–73.7]	26.3	[20.1–33.6]
When you are vaccinated, you protect the institution’s residents	73.7	[68.2–78.6]	25.9	[19.6–33.2]
Perceived barriers to accepting vaccination				
Getting the vaccine is expensive	17.1	[13.3–21.7]	8.9	[4.2–17.8]
The flu vaccine is ineffective	33.4	[28.5–38.7]	7.1	[4.3–11.4]
Avoid the flu vaccine because it causes serious side effects	26.9	[23.4–30.6]	4.8	[2.3–9.7]
The seasonal flu vaccine is not recommended by my doctor	6.1	[4.3–8.5]	3.7	[0.5–22.4]
Getting vaccinated is taking too long	0.4	[0.1–1.5]	0.0	
The promotion of the vaccine is only linked to financial interests	24.1	[20.1–28.5]	4.3	[1.8–9.7]

#### Perceived barriers to influenza vaccination

Table 5 shows that 33.4% of NH workers thought that the influenza vaccine is ineffective and 24.1% of NH workers believed that the promotion of the vaccine was only linked to financial interests. Finally, 26.9% NH workers were convinced that seasonal IV causes serious side effects and 17.1% cited financial costs as a barrier.

## Discussion

The French Ministry of Health has set a 75% target for seasonal IV coverage among NH workers; however, our study estimates the 2015–2016 seasonal IV coverage in Ille-et-Vilaine, in this population, to be 20.0% [15.3%–26.4%]. Vaux et al. [19] reported a 33.6% IV coverage in French NH workers for the 2007–2008 season. Contrary to our study, their study was also showing that private NHs had higher coverage rates than public ones (45.2% versus 29.8%, *p* < 0.001). However, Vaux et al. [19] used data declared by NH management and not direct NH workers interviews, which may introduce bias and prevent the identification of factors linked to individual perception and knowledge.

Social influence may explain the high IV coverage heterogeneity observed in our NH sample, from 0% to 69%, which is consistent with previous studies showing that social influence plays a major role in the decision of vaccination. Studies have found that having a relationship (being a colleague or relative) with people who receive or recommend influenza vaccination is a factor associated to being vaccinated against influenza (*p* < 0.012). Having a NH director or/and NH physician highly invested in the promotion of IV, has also a strong positive impact on NH workers vaccination (*p* < 0,001) [14, 20, 21].

Regarding occupational determinants, we show a significantly higher coverage in administrative staff compared to HCW. This may be explained by the fact that different staff may be sensitive to different kinds of information. We observe strong variations by occupation concerning the preferred communication medium: posters for most of the administrative staff (53.7%) and meetings for most HCW (51.9%). Presently, IV campaigns in French NHs seem to be inefficient; indeed, knowing that a campaign occurred is not significantly link to IV. Looking at individual score of knowledge and perception, our study illustrates a major paradox: firstly, we show that knowledge about means of preventing influenza transmission is highly associated with seasonal IV. Most personnel knew that influenza infection could be avoided by washing their hands, wearing masks and gloves, or avoiding contact with infected people. Of note, “homeopathic vaccine” was also cited by close to 10% of personnel and was proposed by some facilities as an alternative to the real vaccine. In addition, 52.9% of the participants knew that being vaccinated could prevent them from being sick (half of the non-vaccinated), and 73.7% stated that their own vaccination could protect NH residents (two thirds of the non-vaccinated employees). These figures showed that improved education of personnel may play a role in promoting vaccination.

Communication that counters inaccurate beliefs may also need to be specifically implemented. In our study, a third of NH workers interviewed believed that influenza vaccine was ineffective and 26.9% said that it should be avoided due to its side effects. Recent influenza related epidemic events, such as the scare in 2004 of an avian A/H5N1 influenza pandemic, or more recently, the 2009–2010 A/H1N1 pandemic, may have led to influenza communication fatigue or desensitization [20]]. In the Looijmans study, media attention to avian influenza appeared as a positive factor for vaccination (OR 2.24, 95% CI 1.12–4.50), contrary to the 2009–2010 pandemic that did not show any effect [22, 23]. Mass media coverage and health authorities’ communication may have been unable to counter polemics and rumors spread via other channels. For example, the influence of social networks in propagating ideas concerning vaccination has recently been shown [24], which can be considered as a potential issue for health authorities. Receiving information about the effectiveness of the vaccine through an informational meeting and from a nursing home physician has been shown to have a positive impact in different countries [15, 19, 20]. Nevertheless, despite a low IV coverage, NH workers felt they were sufficiently informed, 76.3% by the NHs and 67.5% by the media. Others means of communication have also proven their efficacy like personal reminders [25], newsletters [12], electronic mails, or dedicated websites [26]. However, being vaccinated was associated with having previously experienced a “severe” influenza episode in the past (PR 1.48, 95% CI 1.01–2.17) which shows the differential impact between theoretical and practical knowledge of influenza infection consequences.

Recently, Corace et al. concluded that the Health Belief Model was a promising tool to measure the impact of behavioral changes on the increase of IV coverage among HCW [14]. Perceived benefits and barriers are modifying variables used in the Health Belief Model; however, other variables of this model were not taken into consideration in our study as we did not aim to investigate this concept more deeply. Thus, further studies need to be conducted to explore the outlooks regarding to perceived threats, self-efficacy, as well as cues to actions in order to increase IV uptake.

While being the first study in France to directly interview such a sample of NH workers regarding knowledge and opinions about IV, our study was subject to some limitations. NH participation rate was high (2 refusals out of 40 NHs), and NH workers refusal rate was limited to 1%, but data collection was constrained by the fact that questionnaires had to be administered face-to-face during a single day. In addition, due to logistical issues, our study was performed in a single French department of Brittany (Ille-et-Vilaine). However, potential socio-demographic disparities within Brittany are unlikely to dramatically bias our results. Representativeness of Brittany with regards to France concerning NH workers vaccination is unknown, but to our knowledge, no known characteristics of the Region would impair our estimates at the national level. Although face-to-face interviews are an added value to our study, interviewers could have involuntarily influenced answers of NH workers, a bias minimized by interviewers’ training. Finally, as the subject of this study may have been sensitive to some or all, approaching personal beliefs on vaccination might have made the personnel answer differently from their true opinions.

Despite these limitations, we show that access to vaccination is not likely to be a determinant of low IV coverage, since only 0.4% of the responders declared being vaccinated as time consuming and considering that 80% of the vaccinations occurred inside the facilities. In addition, although 17.1% of the respondents cited IV financial cost as a barrier, the vaccine is available for free (or reimbursed) for the recommended population. Hence, we may “only” face a vaccination “hesitancy” issue, a concern defined by WHO as a “delay in acceptance or refusal of vaccines despite the availability of vaccine services” [27, 28]. Addressing vaccine “hesitancy” is complex and requires targeting multiple potential determinants.

Since most of the personnel vaccination was performed inside NHs like most of the vaccine communication, improvements may have to be targeted at the work place. Indeed, better results are found with IV campaign using staff of mobile units [26], flexible day and time (OR 1.45 95% CI 1.12–1.96) [12], and additional time slots during the day and night, 68.8% versus 41.4% [1].

The next step after influenza vaccine facilitation may be making it mandatory. Compulsory reassignment, wearing a mask or unpaid leave from work in case of refusal to get vaccinated are also different policies associated with a higher IV coverage (*p* < 0.002) [29]. In Norway, public opinion is in favor of freedom of choice (69% among vaccinated, 92.4% among non-vaccinated) [30], so is the Dutch population (77.6% and 96.5% respectively) [20]], but in the US, several hospitals have already made seasonal IV mandatory for HCWs with direct patient contact [15, 31]. Pre-post studies indicate that mandatory vaccination is successful in achieving near-universal vaccination rates of 95% to 98.5% [32], an idea defended by public health specialists for ethical and financial benefits reasons [33–35]. Since NHs have a moral responsibility to protect their residents, NH workers mandatory IV may have to be considered in the future if IV coverage remains low due to inefficiency of voluntary incitation measures.

## Conclusions

Our study showed that NH workers IV maybe largely insufficient in western France and possibly throughout the country. High variations in IV coverage among NHs shows the key role of NH management and the working environment. Vaccine “hesitancy” may also be the main reason explaining this public health issue. Specific communication tools focusing on false beliefs should be developed and adapted to the various professional population, as well as the new modes of communication (social media). Failing to do so may pave the way to more debatable strategies such as compulsory IV.

## Additional files

Additional file 1: Nursing home questionnaire. (DOCX 18 kb)

Additional file 2: Questionnaire EHPAD. (DOCX 18 kb)

Additional file 3: Nursing home workers questionnaire. (DOCX 20 kb)

Additional file 4: Questionnaire personnel permanent EHPAD. (DOCX 24 kb)

## Abbreviations

ANSP: Santé Publique France
ARS: Agence Régionale de Santé
CDC: Center for Disease Control and Prevention
CESPA: Centre d’épidémiologie et de santé publique des armées
CI: Confidence interval
EHESP: French School of Public Health
HCW: Healthcare workers
IDEA: International field epidemiology training course
IV: Influenza vaccination
NHs: Nursing homes
OR: Odds ratio
PR: Prevalence ratio
WHO: World Health Organization

## References

[1] Centers for Disease Control and Prevention (CDC). Influenza vaccination coverage among health-care personnel --- United States, 2010–11 influenza season. MMWR Morb Mortal Wkly Rep. 2011;60(32):1073–7. 21849963

[2] Vaccines against influenza WHO position paper – November 2012. Relevé Épidémiologique Hebd Sect Hygiène Secrétariat Société Nations Wkly Epidemiol Rec Health Sect Secr Leag Nations. 2012;87(47):461–76.

[3] Hak E, Wei F, Nordin J, Mullooly J, Poblete S, Nichol KL (2004). Development and validation of a clinical prediction rule for hospitalization due to pneumonia or influenza or death during influenza epidemics among community-dwelling elderly persons. J Infect Dis.

[4] Jefferson T, Rivetti D, Rivetti A, Rudin M, Di Pietrantonj C, Demicheli V (2005). Efficacy and effectiveness of influenza vaccines in elderly people: a systematic review. Lancet Lond Engl.

[5] Hayward AC, Harling R, Wetten S, Johnson AM, Munro S, Smedley J (2006). Effectiveness of an influenza vaccine programme for care home staff to prevent death, morbidity, and health service use among residents: cluster randomised controlled trial. BMJ.

[6] van den Dool C, Bonten MJM, Hak E, Heijne JCM, Wallinga J (2008). The effects of influenza vaccination of health care workers in nursing homes: insights from a mathematical model. PLoS Med.

[7] Mitchell R, Huynh V, Pak J, Thompson S, Noseworthy AL (2006). Influenza outbreak in an Ontario long-term care home--January 2005. Can Commun Dis Rep Relevé Mal Transm Au Can.

[8] Bush KA, McAnulty J, McPhie K, Reynolds R, Boomer M, Clarkson LM (2004). Antiviral prophylaxis in the management of an influenza outbreak in an aged care facility. Commun Dis Intell Q Rep.

[9] Wilde JA, McMillan JA, Serwint J, Butta J, O’Riordan MA, Steinhoff MC (1999). Effectiveness of influenza vaccine in health care professionals: a randomized trial. JAMA.

[10] National Vaccine Advisory Committee. Strategies to achieve the healthy people 2020 annual influenza vaccine coverage goal for health-care personnel: recommendations from the national vaccine advisory committee. Public Health Rep Wash DC 1974. 2013;128(1):7–25. PMC3514716 23277655

[11] Loi n° 2004–806 du 9 août 2004 relative à la politique de santé publique - Article ANNEXE.

[12] Pina P, Moreau AL, Duran A, Sadeg O, Mandelbaum B, Teixeira A (2008). Acceptance of influenza vaccination by caregivers in a geriatric and long-term care institution. Médecine Mal Infect.

[13] LaVela SL, Etingen B, Miskevics S (2015). Attitudes toward influenza vaccination improvement strategies in veterans affairs health care workers providing care for patients with spinal cord injuries and disorders: acceptability of a declination form program. Vaccine.

[14] Corace K, Prematunge C, McCarthy A, Nair RC, Roth V, Hayes T (2013). Predicting influenza vaccination uptake among health care workers: what are the key motivators?. Am J Infect Control.

[15] Daugherty JD, Blake SC, Grosholz JM, Omer SB, Polivka-West L, Howard DH (2015). Influenza vaccination rates and beliefs about vaccination among nursing home employees. Am J Infect Control.

[16] Mehta M, Pastor CA, Shah B (2008). Achieving optimal influenza vaccination rates: a survey-based study of healthcare workers in an urban hospital. J Hosp Infect.

[17] Vaux S, Noël D, Fonteneau L, Guthmann J-P, Lévy-Bruhl D (2010). Influenza vaccination coverage of healthcare workers and residents and their determinants in nursing homes for elderly people in France: a cross-sectional survey. BMC Public Health.

[18] Guthmann J-P, Fonteneau L, Ciotti C, Bouvet E, Pellissier G, Lévy-Bruhl D (2012). Vaccination coverage of health care personnel working in health care facilities in France: results of a national survey, 2009. Vaccine.

[19] Vaux S, Van Cauteren D, Guthmann J-P, Le Strat Y, Vaillant V, de Valk H (2011). Influenza vaccination coverage against seasonal and pandemic influenza and their determinants in France: a cross-sectional survey. BMC Public Health.

[20] Looijmans-van den Akker I, van Delden JJM, Verheij TJM, van Essen GA, van der Sande MA, Hulscher ME (2009). Which determinants should be targeted to increase influenza vaccination uptake among health care workers in nursing homes?. Vaccine.

[21] Takayanagi IJ, Cardoso MRA, Costa SF, Araya MES, Machado CM (2007). Attitudes of health care workers to influenza vaccination: why are they not vaccinated?. Am J Infect Control.

[22] Guthmann J-P, Fonteneau L, Bonmarin I, Lévy-Bruhl D (2012). Influenza vaccination coverage one year after the a(H1N1) influenza pandemic, France, 2010–2011. Vaccine.

[23] Bone A, Guthmann J-P, Nicolau J, Lévy-Bruhl D (2010). Population and risk group uptake of H1N1 influenza vaccine in mainland France 2009–2010: results of a national vaccination campaign. Vaccine.

[24] Salathé M, Khandelwal S (2011). Assessing vaccination sentiments with online social media: implications for infectious disease dynamics and control. PLoS Comput Biol.

[25] Lee SJ, Harrison R, Rosenberg J, McLendon P, Boston E, Lindley MC (2013). Influenza vaccination among health care personnel in California: 2010–2011 influenza season. Am J Infect Control.

[26] Llupià A, García-Basteiro AL, Olivé V, Costas L, Ríos J, Quesada S (2010). New interventions to increase influenza vaccination rates in health care workers. Am J Infect Control.

[27] WHO. Report of the Sage Working Group on Vaccine Hesitancy. 2014.

[28] ECDC. European Centre for Disease Prevention and Control. Vaccine hesitancy among healthcare workers and their patients in Europe – A qualitative study. 2015.

[29] Nowalk MP, Lin CJ, Raymund M, Bialor J, Zimmerman RK (2013). Impact of hospital policies on health care workers’ influenza vaccination rates. Am J Infect Control.

[30] Bentele H, Bergsaker MR, Hauge SH, Bjørnholt JV (2014). Vaccination coverage for seasonal influenza among residents and health care workers in Norwegian nursing homes during the 2012/13 season, a cross-sectional study. BMC Public Health.

[31] Stewart AM, Cox MA (2013). State law and influenza vaccination of health care personnel. Vaccine.

[32] Septimus EJ, Perlin JB, Cormier SB, Moody JA, Hickok JD (2011). A multifaceted mandatory patient safety program and seasonal influenza vaccination of health care workers in community hospitals. JAMA.

[33] Poland GA (2010). Mandating influenza vaccination for health care workers: putting patients and professional ethics over personal preference. Vaccine.

[34] Ottenberg AL, Wu JT, Poland GA, Jacobson RM, Koenig BA, Tilburt JC (2011). Vaccinating health care workers against influenza: the ethical and legal rationale for a mandate. Am J Public Health.

[35] van Delden JJM, Ashcroft R, Dawson A, Marckmann G, Upshur R, Verweij MF (2008). The ethics of mandatory vaccination against influenza for health care workers. Vaccine.

