# Supplementary material for: Seasonal influenza vaccination coverage and its determinants among nursing homes personnel in western France
Source: BMC Public Health. 2017 Jul 7;17:634. doi: 10.1186/s12889-017-4556-5 (PMC5501011; doi:10.1186/s12889-017-4556-5)
Supplement: Supplementary file 1 — Nursing home questionnaire. (DOCX 18 kb) [file 12889_2017_4556_MOESM1_ESM.docx]

**Nursing home questionnaire**

**Nursing home (NH):** _____________ **Interviewer:** _______________ **Number of questionnaire:** _____________

1. **Name of the NH**: _________________________________________________________________________
2. **Location**: ________________________________________________________________________________
3. **Nursing home status**:

□ Public □ Private

1. **Size of the nursing home**:

Number of residents:_________________________________________________________________

Number of NH workers: ______________________________________________________________

1. **Dependence score**: ________________________________________________________________________
2. **Time of visit**: ____ H____
3. **Number of NH workers present at the time of the survey**: _______________________________________
4. **For the season 2015/2016, in the NH, there was**:

□ a NH workers sensitization

□ a vaccination campaign

1. **If there was a vaccination campaign, did it took place inside the NH ?**

□ Yes □ No
