# Supplementary material for: Seasonal influenza vaccination coverage and its determinants among nursing homes personnel in western France
Source: BMC Public Health. 2017 Jul 7;17:634. doi: 10.1186/s12889-017-4556-5 (PMC5501011; doi:10.1186/s12889-017-4556-5)
Supplement: Supplementary file 2 — Questionnaire EHPAD. (DOCX 18 kb) [file 12889_2017_4556_MOESM2_ESM.docx]

**Questionnaire EHPAD**

**Etablissement :** _____________ **Enquêteur:** _______________ **Numéro d’ordre:** _____________

1. **Nom de l’établissement** :___________________________________________________________________
2. **Lieu**: ___________________________________________________________________________________
3. **Statut de l’établissement**:

□ Public □ Privé

1. **Taille de l’établissement**:

Nombre de résidents accueillis : _______________________________________________________

Nombre de personnel permanent: _______________________________________________________

1. **Score de dépendance (GMP 2016)**: __________________________________________________________
2. **Heure de la visite** : ____ H____
3. **Nombre de personnel permanent au moment de l’enquête** : _____________________________________
4. **Pour la saison 2015/2016, dans l’EHPAD, il y a eu**:

□ une sensibilisation du personnel

□ une campagne de vaccination

1. **S’il y a eu une campagne de vaccination, a-t’ elle eu lieu sur place?**

□ Oui □ Non
