# Supplementary material for: Seasonal influenza vaccination coverage and its determinants among nursing homes personnel in western France
Source: BMC Public Health. 2017 Jul 7;17:634. doi: 10.1186/s12889-017-4556-5 (PMC5501011; doi:10.1186/s12889-017-4556-5)
Supplement: Supplementary file 3 — Nursing home workers questionnaire. (DOCX 20 kb) [file 12889_2017_4556_MOESM3_ESM.docx]

**Nursing home workers questionnaire**

**Nursing home (NH):** _____________ **Interviewer:** _______________ **Number of questionnaire:** _____________

Hello, my name is …, I am an epidemiologist and I am currently conducting a survey, following the request of our regional health authority of Brittany and Santé Publique France, concerning the seasonal influenza vaccination coverage among NH workers. Do you agree to answer to this questionnaire? It will not last more than five minutes.

1. **Gender** : □ M □ F
2. **How old are you?** : ________________________________________________________________________
3. **What is your profession?**

□ Physician □ Reception agent

□ Nurse □ Maintenance staff

□ Pharmacist □ Cooking staff

□ Caregiver □ Animation staff

□ Administration staff □ Other : _______________________________________________

1. **How many years have you been working in a NH**: ______________________________________________
2. **How often would you consider being close to less than one meter with a resident?**

□ Never □ Once a day

□ Less than once a week □ More than once a day

□ Once or more than once a week

1. **Among people living with you, are there any :**

□ Children aged 5or below □ Person having health troubles that justify to have a flu shot

□ Person aged 65 or above

1. **Have you had a flu shot (by injection) since October 1^st^, 2015?**

□ Yes □ No

- 1. **If yes, who vaccinated you?**

□ General practitioner □ Occupational Physician

□ NH Occupational Physician

1. **Did we suggest you a flu shot because of health disorders (non-communicable diseases, pregnancy)?**

□ Yes □ No

1. **During the past 3 years (winter 2013), how many have you had a flu shot? ___________________________**
2. **Have you ever had a severe episode of influenza in the past life?**

□ Yes □ No

1. **According to you, how would you avoid flu?**

□ By vaccination □ By taking homeopathy

□ By washing hands □ Other: ________________________________________

□ By taking antivirals □ Don’t know

□ By wearing masks and gloves

1. **According to you, what are the populations most a risk to die from the flu?**

□ Person aged 65 or above □ Persons with non-communicable diseases

□ Infants □ Other : ________________________________________

□ Pregnant women □ Don’t know

□ Overweight persons

1. **According to you, how often a NH worker has to get a flu shot?**

□ Every year □ Every three years or more

□ Every 2 years □ Don’t know

1. **This season 2015-2016, did you receive any information concerning vaccination against influenza?**

□ By the NH □ By an occupational practitioner

□ By the media □ By another source of information

□ By your GP

1. **Did this information influence your choice of being vaccinated or not?**

□ Yes □ No

1. **Did you consider being sufficiently informed within your NH?**

□ Yes □ No

1. **What kind of information would you be sensitive to ?**

□ Billboards □ Meetings, courses

□ Emails/mails/notes □ Other : _______________________________________________

1. **During the season 2015-2016, was any vaccination campaign against influenza organized by the NH?**

□ Yes □ No

1. **Among this suggestions, please tell us if you agree or disagree :**

|  | **Agree** | **Disagree** |
| --- | --- | --- |
| Being vaccinated protects you against influenza |  |  |
| Being vaccinated is expensive |  |  |
| Influenza vaccine is inefficient |  |  |
| Being vaccinated protects your entourage |  |  |
| Being vaccinated protects NH residents |  |  |
| You have to avoid being vaccinated because of its serious side effects |  |  |
| Getting a vaccine takes a long time |  |  |
| Vaccine promotion is only linked to financial interests |  |  |

Did you have any suggestions or remarks?

We thank you very much for your participation. A feedback will be sent to your NH as soon as we get all the results.
