# Supplementary material for: Seasonal influenza vaccination coverage and its determinants among nursing homes personnel in western France
Source: BMC Public Health. 2017 Jul 7;17:634. doi: 10.1186/s12889-017-4556-5 (PMC5501011; doi:10.1186/s12889-017-4556-5)
Supplement: Supplementary file 4 — Questionnaire personnel permanent EHPAD. (DOCX 24 kb) [file 12889_2017_4556_MOESM4_ESM.docx]

**Questionnaire personnel permanent EHPAD**

**Etablissement :** _____________ **Enquêteur:** _______________ **Numéro d’ordre:** _____________

Bonjour, je m’appelle …, je suis épidémiologiste et mène une enquête à la demande de l’ARS Bretagne et Santé Publique France sur la couverture vaccinale antigrippe saisonnière des personnels travaillant en EHPAD. Acceptez-vous de répondre à ce questionnaire qui vous prendra environ 5 minutes ?

1. **Sexe** : □ M □ F
2. **Quel âge avez-vous?** : ________________________________________________________________________
3. **Quelle est votre profession?**

□ Médecin □ Agent d’accueil

□ Infirmière □ Personnel d’entretien

□ Pharmacien □ Personnel de restauration

□ Aide-soignant □ Personnel d’animation

□ Personnel administratif □ Autre : _______________________________________________

1. **Combien d’années avez-vous travaillé en EHPAD ?**: ______________________________________________
2. **A quelle fréquence vous trouvez-vous à moins d’un mètre d’un résident ?**

□ Jamais □ Une fois par jour

□ Moins d’une fois/semaine □ Plus d’une fois par jour

□ Une ou plusieurs fois par semaine

1. **Parmi les personnes vivant avec vous, y a-t-il :**

□ Enfant de moins de 5 ans □ Personne avec des problèmes de santé justifiant une vaccination

□ Personne âgée de 65 ans ou plus antigrippale

1. **Avez-vous été vacciné contre la grippe par un vaccin injectable depuis le 1^er^ Octobre 2015?**

□ Oui □ Non

- 1. **Si oui, par qui avez-vous été vacciné ?**

□ Médecin traitant □ Médecin du travail hors EHPAD

□ Médecin du travail EHPAD

1. **Vous a-t-on proposé la vaccination antigrippale pour des raisons de santé (pathologies chroniques, grossesse) ?**

□ Oui □ Non

1. **Durant ces 3 dernières années (hiver 2013), combien de fois vous êtes-vous fait vacciner contre la grippe? _**
2. **Avez-vous déjà eu une grippe qui vous a cloué au lit?**

□ Oui □ Non

1. **Selon vous, comment éviter la grippe ?**

□ Par la vaccination □ Avec de l’homéopathie

□ En se lavant les mains □ Autre : ________________________________________

□ En prenant de antiviraux □ Ne sait pas

□ En portant un masque et des gants

1. **Selon vous, quelles sont les populations les plus à risque de mourir de la grippe?**

□ Enfants en bas âge □ Personnes avec de pathologies chroniques

□ Femmes enceintes □ Autres : ________________________________________

□ Personnes en surpoids □ Ne sait pas

□ Personne âgée de 65 ans ou plus

1. **Selon vous, à quelle fréquence un personnel d’EHPAD doit-il se faire vacciner?**

□ Chaque année □ Tous les 3 ans ou plus

□ Tous les 2 ans □ Ne sait pas

1. **Cette saison 2015-2016, avez-vous reçu des informations sur la vaccination antigrippale ?**

□ Par l’EHPAD □ Par le médecin du travail

□ Par les médias □ Par une autre source d’information

□ Par votre médecin traitant

1. **Ces informations ont-elles influencé votre choix de vaccination?**

□ Oui □ Non

1. **Pensez- vous avoir été suffisamment informé au sein de votre établissement ?**

□ Oui □ Non

1. **A quels types d’informations seriez-vous sensible ?**

□ Affiches □ Réunions d’informations, formations

□ Emails/courrier/note □ Autre : _______________________________________________

1. **Courant la saison 2015-2016, une campagne de vaccination a-t-elle été organisée par l’EHPAD?**

□ Oui □ Non

1. **Parmi ces propositions, dites si vous êtes plutôt d’accord ou pas d’accord :**

|  | **D’accord** | **Pas d’accord** |
| --- | --- | --- |
| Le vaccin vous protège contre la grippe |  |  |
| Se faire vaccine coute cher |  |  |
| Le vaccin antigrippe est inefficace |  |  |
| Quand vous êtes vacciné, vous protégez votre entourage |  |  |
| Quand vous êtes vacciné, vous protégez les résidents de l’EHPAD |  |  |
| Il faut éviter le vaccin antigrippe à cause de ses effets secondaires graves |  |  |
| Se vacciner prend trop de temps |  |  |
| La promotion du vaccin n’est liée qu’à des intérêts financiers |  |  |

Avez-vous des suggestions ou des remarques?

Nous vous remercions pour votre participation. Une rétro-information sera communiquée auprès de votre établissement .
